# The role of serum biomarkers in the decline of cognitive function in hemodialysis patients: a single-center retrospective study

**DOI:** 10.3389/fmed.2025.1556584

**Published:** 2025-08-29

**Authors:** Yulu Li, Riyue Jiang, Bin Zhu, Jie Yu, Liying Miao, Jianqin Shen

**Affiliations:** ^1^Department of Nephrology, Taicang Loujiang New City Hospital, Suzhou, China; ^2^Department of Radiation Oncology, The First Affiliated Hospital of Nanjing Medical University, Nanjing, China; ^3^Department of Critical Care Medicine, The Third Affiliated Hospital of Soochow University, Changzhou, China; ^4^Department of Nephrology, The Third Affiliated Hospital of Soochow University, Changzhou, China; ^5^Blood Purification Centre, The Third Affiliated Hospital of Soochow University, Changzhou, China

**Keywords:** hemodialysis, cognitive decline, serum biomarkers, risk factors, quality of life

## Abstract

**Background:**

Cognitive impairment in hemodialysis patients is higher than that in healthy individuals. However, the potential pathogenesis is not fully clear.

**Methods:**

In this retrospective study, 48 maintenance hemodialysis (MHD) patients of our center who participated in cognitive level test 4 years ago were screened by Montreal Cognitive Assessment (MoCA). We then analyzed relationships between the MoCA-derived cognitive level score, demographic, clinical, and laboratory variables.

**Results:**

The age, dialysis age, Body Mass Index (BMI), and blood calcium levels of MHD patients were significantly abnormal than 4 years ago in this study. Furthermore, we observed that the levels of fibroblast growth factor (FGF-23) in MHD patients with cognitive decline were significantly higher than those in the normal cognitive group. Receiver operating characteristic (ROC) curves demonstrated that FGF-23 was a potential biomarker for diagnosing cognitive decline in hemodialysis patients.

**Conclusion:**

We speculate that dialysis age, increased BMI, decreased blood calcium, and the abnormal level of FGF-23 may be independent risk factors for cognitive impairment in hemodialysis patients. Moreover, BMI and decreased blood calcium can be used as predictive factors of hemodialysis-related cognitive impairment.

## Introduction

Chronic kidney disease (CKD) is now recognized as a major global public health problem. The global prevalence of CKD gradually rises, reaching up to 13.4% (1, 2). In CKD patients, the interaction between the kidney and brain can lead to various nervous system diseases, including both functional and organic conditions such as cerebrovascular disease, cognitive impairment, and autonomic and peripheral nervous system diseases (3). Notably, there is a growing focus on the cognitive impairment seen in CKD patients.

Cognition, which refers to the acquisition and application of knowledge, is a fundamental mental process that involves various aspects such as memory, attention, language, time–space orientation, execution, calculation, and judgment. Therefore, cognitive impairment can significantly impact the daily activities of individuals, ranging from mild to severe levels of impairment. It is a crucial function of the brain that can be affected by various factors. One of these factors is chronic kidney disease, which contributes to cognitive impairment through hypertension caused by renal dysfunction, over-activated renin-angiotensin system (RAS), renal anemia, renal osteopathy, and small vessel damage (4). Indeed, the incidence of cognitive impairment in CKD patients receiving maintenance hemodialysis (MHD) has been reported to be as high as 80% (5, 6). Therefore, CKD is considered one of the strongest risk factors for cognitive impairment. This impairment greatly affects MHD patients in terms of decision-making capacity, self-care ability, and treatment compliance, increasing the occurrence of negative emotions and the risk of death (7, 8). However, there is currently a lack of literature that tracks and re-evaluates hemodialysis patients with cognitive impairment, and knowledge about the potential pathogenesis of cognitive impairment in these patients remains limited.

In 2018, a single-center study was conducted to evaluate the cognitive function in 58 CKD patients (9). The results of this study showed that the cognitive function of MHD patients was significantly worse compared to that of healthy controls. Additionally, it was observed that MHD patients with cognitive impairment exhibited significantly lower levels of brain-derived neurotrophic factor (BDNF) and platelet counts (PLT) in comparison with both healthy controls and individuals with normal cognitive function. On the other hand, the levels of tumor necrosis factor (TNF)-α and interleukin 6 (IL-6) were significantly higher in MHD patients with cognitive impairment. These findings suggest that abnormalities in BDNF, TNF-α, IL-6, and PLT counts in serum may be associated with hemodialysis-related cognitive impairment.

In this study, we aimed to investigate the correlation between abnormalities found in patients 4 years ago and cognitive decline. By following up with the participants who were part of the study 4 years ago, we sought to identify the factors that exhibited abnormalities and examine their association with cognitive decline. This investigation may provide valuable insights into potential indicators for early intervention, which could prove beneficial in preventing cognitive decline in MHD patients.

## Methods

### Participants and study design

This is a retrospective study. In February to May 2017, we selected 58 patients undergoing hemodialysis at the Blood Purification Centre, The Third Affiliated Hospital of Soochow University (9). Out of these patients, 42 were included in this study. The study group consisted of 21 males and 21 females, with an average age of 49.07 ± 7.3 years. The primary diseases observed were chronic glomerulonephritis, type II diabetes, type I diabetes, benign arteriolosclerosis, polycystic kidney disease, nephrotic syndrome, medullary sponge kidney, and systemic lupus erythematosus in 23, 2, 1, 1, 2, 3, 1, and 1 patients, respectively. The remaining eight patients had an unknown cause of CKD. Comparatively, 16 individuals from the original cohort did not participate in the follow-up. Among these, three patients had been transferred, five had died, seven had undergone kidney transplants, and one did not participate due to vision problems.

Exclusion criteria were history of surgery for invasive intracranial pressure monitoring; mental or neurological diseases such as dementia, Alzheimer’s disease and schizophrenia; brain trauma; use of antiepileptic drugs, alcohol, or illicit drugs. Written informed consent was obtained from all participants, and the study was approved by the Ethics Commission of Soochow University (Changzhou, China) and conducted in accordance with the principles of Declaration of Helsinki. This clinical study was registered with the Chinese Clinical Trial Register (Clinical Trial Number: ChiCTR-ROC-16009283).

### Treatment

All patients enrolled in this study underwent blood purification three times a week. The treatment regimen was hemodialysis once a week combined with hemodiafiltration twice a week, or hemodialysis twice a week combined with hemodiafiltration once a week. A Fresenius low-throughput polysulfone membrane dialyser (Fresenius FX8, Germany) was used for hemodialysis, and the hemodiafiltration was performed using a Fresenius high-throughput polysulfone membrane dialyser (Fresenius FX80, Germany). Each session was about 4 h, and low-molecular-weight heparin was used for anticoagulation. The dialysis solution was bicarbonate, the average blood flow rate is 200–280 mL/min, and the dialysis flow rate was 500 mL/min. The displacement volume after replacement was about 30% of ultrafiltration flow rate.

### Cognitive function tests

Montreal Cognitive Assessment (MoCA) is a test to evaluate the cognitive function of patients. The test concerns eight cognitive domains: concentration, executive function, memory, linguistic ability, the capacity of visual space, abstract thought, computing power, and orientation. For those whose educational level is 12 or fewer years, one point will be added. The total score is 30 points. A score of MoCA ≥26 is considered to indicate normal cognition, and the score < 26 is considered to indicate cognitive impairment.

### Laboratory tests

All patients’ blood were examined before hemodialysis within 1 week of cognitive function test. Leukocyte count, red blood cell count, hemoglobin, platelet, and hematocrit were measured using an automated five-class blood cell analyzer electrical impedance device (Sysmex Corporation, Japan). Blood urea nitrogen, creatinine, calcium, phosphorus, and albumin were detected by an automated biochemical analyzer (Leadman Biochemical, China). Parathyroid hormone measurement was achieved using chemiluminescence (Beckman, United States). Test methods and results for serum α-klotho, fibroblast growth factor (FGF-23), IL-6, BDNF, and tumor necrosis factor (TNF)-α levels were derived from the study conducted our years ago (9).

### Statistical analysis

Data were expressed as means ± standard error of the mean (S.E.M.). Statistical analyses were performed using SPSS 26.0 (SPSS, Chicago, IL, United States). Comparisons between groups were performed by or *χ*2 test, Student’s *t*-test or nonparametric tests. The diagnostic value of test data for diseases was clarified by ROC curve analysis. Survival analysis was used to predict what different levels of the serum biological marker do to the occurrence and development of the disease. A *p* < 0.05 shows a statistically significant difference.

## Results

### Cognitive function is worse in hemodialysis patients after 4 years than those 4 years ago

**Figure 1:**
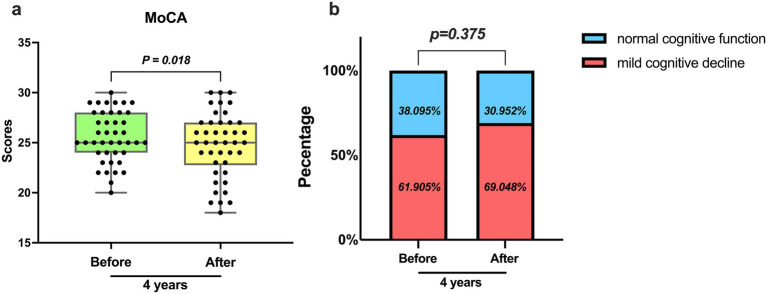
Assessment of cognitive function by MoCA. **(a)** The average level and degree of variation of MoCA scores 4 years ago and 4 years later (*p* = 0.018). **(b)** Occurrence of cognitive impairment 4 years ago and 4 years later (*p* = 0.375).

According to the cognitive function test (MoCA) (Figure 1a), the level of cognitive function was evaluated for CKD patients undergoing hemodialysis 4 years ago and after 4 years. Four years ago, the maximum cognitive score was 30 points, with the upper quarter at 28 points, median at 25 points, lower quarter at 24 points, and the minimum at 20 points. In comparison, 4 years later, the maximum cognitive score remained at 30 points, with the upper quarter decreasing to 27 points, median remaining at 25 points, lower quarter decreasing to 23 points, and the minimum decreasing to 18 points. It was observed that on average, the cognitive scores decreased after 4 years when compared to the scores from 4 years ago, indicating a statistically significant difference. The prevalence of cognitive impairment also increased from 61.9% 4 years ago to 69.0% 4 years later. Therefore, there has been an increase in the proportion of hemodialysis patients experiencing cognitive decline, in comparison with 4 years ago (Figure 1b).

### General clinical characteristics of patients receiving hemodialysis

Table 1 presents the general clinical data of the study patients, encompassing age, sex, routine blood test values, and biomedical parameters. Analyzing the data 4 years ago and 4 years later, we found significant differences in age, dialysis duration, Body Mass Index (BMI), and calcium. Conversely, leukocyte count, red blood cell count, hemoglobin, platelet count, hematocrit, blood urea nitrogen, creatinine, phosphorus, albumin, parathyroid hormone, urea reduction ratio (URR), and Kt/V were not found to be different.

**Table 1:** General characteristics of study participants (*n* = 42).

Factor	Four years ago	Four years later	*t*	*Z*	*p*
Age (years)	45.79 ± 6.85	49.07 ± 7.31	−13.33		<0.001^a^
BMI	21.94 ± 2.37	23.12 ± 2.97		−3.645	<0.001^b^
Hb (g/L)	108.83 ± 15.20	111.05 ± 14.51		−0.607	0.544^b^
Hematocrit (L/L)	0.33 ± 0.05	0.35 ± 0.04		−1.526	0.127^b^
RBC (10^12^/L)	3.63 ± 0.54	3.76 ± 0.53	−1.848		0.072^a^
WBC (10^9^/L)	6.34 ± 1.83	5.66 ± 1.35		−1.913	0.056^b^
PLT (10^9^/L)	184.45 ± 57.08	185.81 ± 63.87		−0.419	0.675^b^
BUN (mmol/L)	25.20 ± 6.48	25.06 ± 5.44		−0.25	0.803^b^
Cr (μmol/L)	946.39 ± 126.18	952.66 ± 139.79		−0.35	0.726^b^
Calcium (mmol/L)	2.38 ± 0.27	2.25 ± 0.20		−3.001	0.003^b^
Phosphate (mmol/L)	2.18 ± 0.63	2.08 ± 0.52		−0.563	0.574^b^
PTH (pg/mL)	483.78 ± 478.62	468.79 ± 423.73		−0.288	0.774^b^
ALB (g/L)	39.68 ± 2.15	39.69 ± 2.57	−0.034		0.973^a^
URR	69.46 ± 3.64	69.21 ± 4.01		−0.369	0.712^b^
Kt/V	1.46 ± 0.16	1.42 ± 0.17		−0.944	0.345^b^
Dialysis duration (months)	80.24 ± 43.37	135.17 ± 42.73	−9.342		<0.001^a^

Clinical and biological parameters of patients between the data 4 years ago and 4 years later. Data are shown as means ± standard error of means. BMI, body mass index; Hb, hemoglobin; RBC, red blood cell; WBC, white blood cell; PLT, blood platelet; ALB, albumin; BUN, blood urea nitrogen; Cr, creatinine; PTH, parathyroid hormone; URR, urea reduction ratio; Kt/V, the urea clearance of the dialyzer during dialysis (K), multiplied by the time (t) of dialysis, divided by the patient’s urea distribution volume (V). The superscript ‘a’ denotes that the data for the study were normally distributed. Conversely, ‘b’ denotes non-normally distributed data.

### Serum biomarker levels

In light of whether their MoCA scores after 4 years had decreased compared to 4 years ago, 42 hemodialysis patients were divided into two groups. The analysis of serum biomarker levels 4 years ago revealed no significant differences in α-klotho, IL6, TNF-α, and BDNF between the two groups. However, a significant difference was observed in FGF-23 (Table 2).

**Table 2:** Serum biomarker levels of study participants (*n* = 17−25).

Serum biomarker	No decline in cognitive function	Cognitive decline	*t*	*Z*	*p*
α-Klotho (pg/mL)	486.47 ± 48.32	501.13 ± 31.79		−0.820	0.412^b^
FGF-23 (ng/mL)	945.14 ± 247.29	1620.02 ± 180.64		−2.179	0.029^b^
IL-6 (pg/mL)	3.29 ± 1.47	1.78 ± 0.24		−0.551	0.581^b^
TNF-α (pg/mL)	30.25 ± 4.17	24.46 ± 1.75		−0.872	0.383^b^
BDNF (ng/mL)	14.01 ± 1.63	12.66 ± 1.43	0.615		0.542^a^

Serum biomarker levels of patients between the data 4 years ago and 4 years later. Data are shown as means ± standard error of means. The superscript ‘a’ denotes that the data for the study were normally distributed. Conversely, ‘b’ denotes non-normally distributed data.

### Evaluation of serum biomarkers, BMI and calcium for the diagnosis of hemodialysis-related cognitive decline using ROC curve analysis

**Figure 2:**
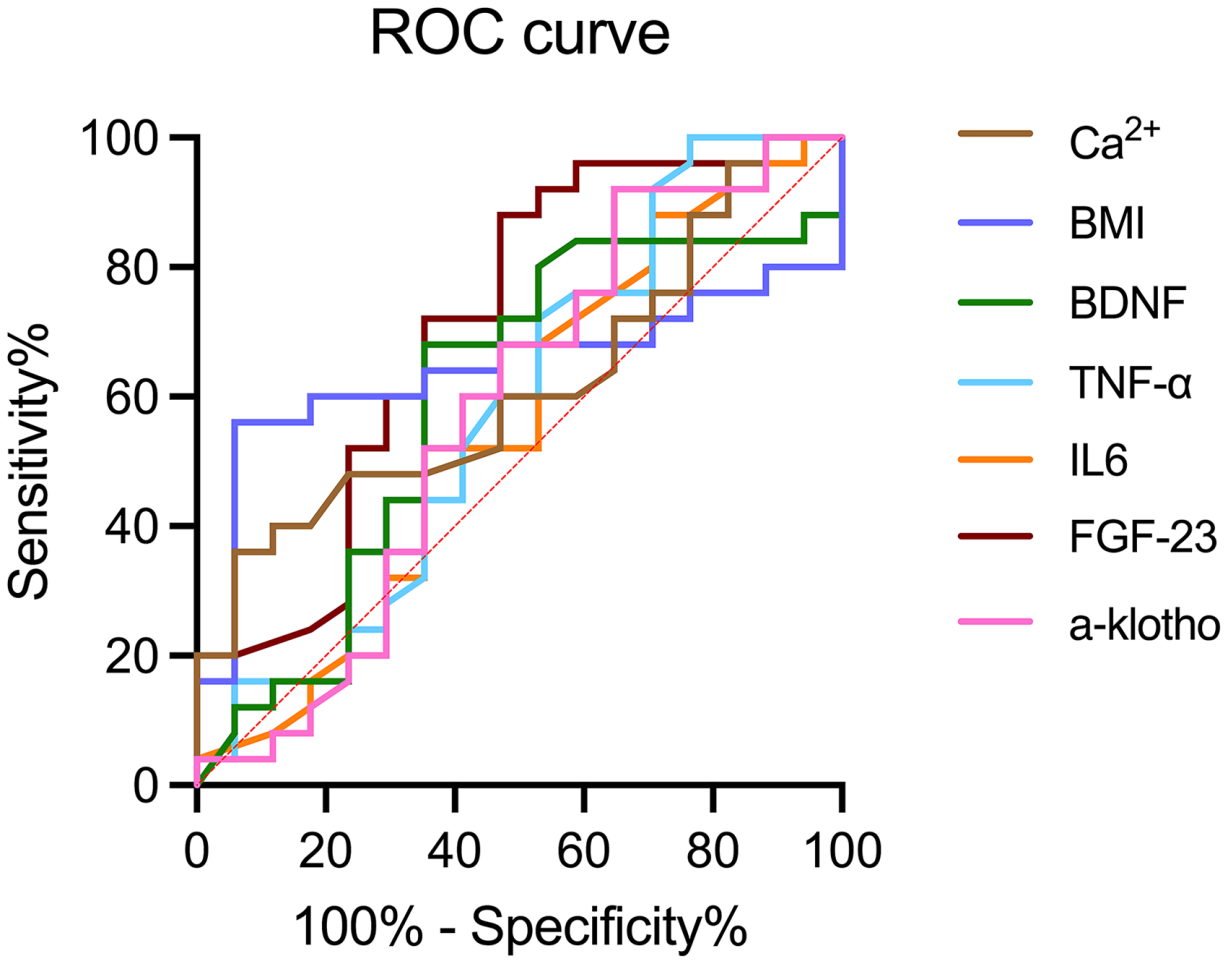
ROC curves of BMI, calcium, and serum biomarkers, including α-klotho, FGF-23, IL-6, TNF-α, and BDNF for the diagnosis of hemodialysis-related cognitive decline. BDNF, brain-derived neurotrophic factor; FGF-23, fibroblast growth factor-23; IL-6, interleukin-6; TNF-α, tumor necrosis factor-α; ROC, receiver operating characteristic.

Analysis of all dialysis-induced cognitive decline cases revealed that the area under the ROC curve for α-klotho, FGF-23, IL-6, TNF-α, BDNF, BMI, and calcium were 0.58, 0.55, 0.58, 0.59, 0.70, 0.64, and 0.62, respectively (Figure 2). The best cutoff values for serum α-klotho, FGF-23, IL-6, TNF-α, BDNF, BMI, and calcium levels for the diagnosis of dialysis-induced cognitive decline were 469.5 pg/mL, 1,264.5 ng/mL, 1.8 pg/mL, 21.1 pg/mL, 20.0 ng/mL, 23.9 kg/m^2^, and 2.52 mmol/L, respectively. In summary, the diagnostic value of serum biomarkers, BMI, and calcium for hemodialysis-related cognitive decline can be summarized.

### Evaluation of the relativity of serum biomarkers, BMI, age, and calcium to hemodialysis-related cognitive decline using survival analysis

**Figure 3:**
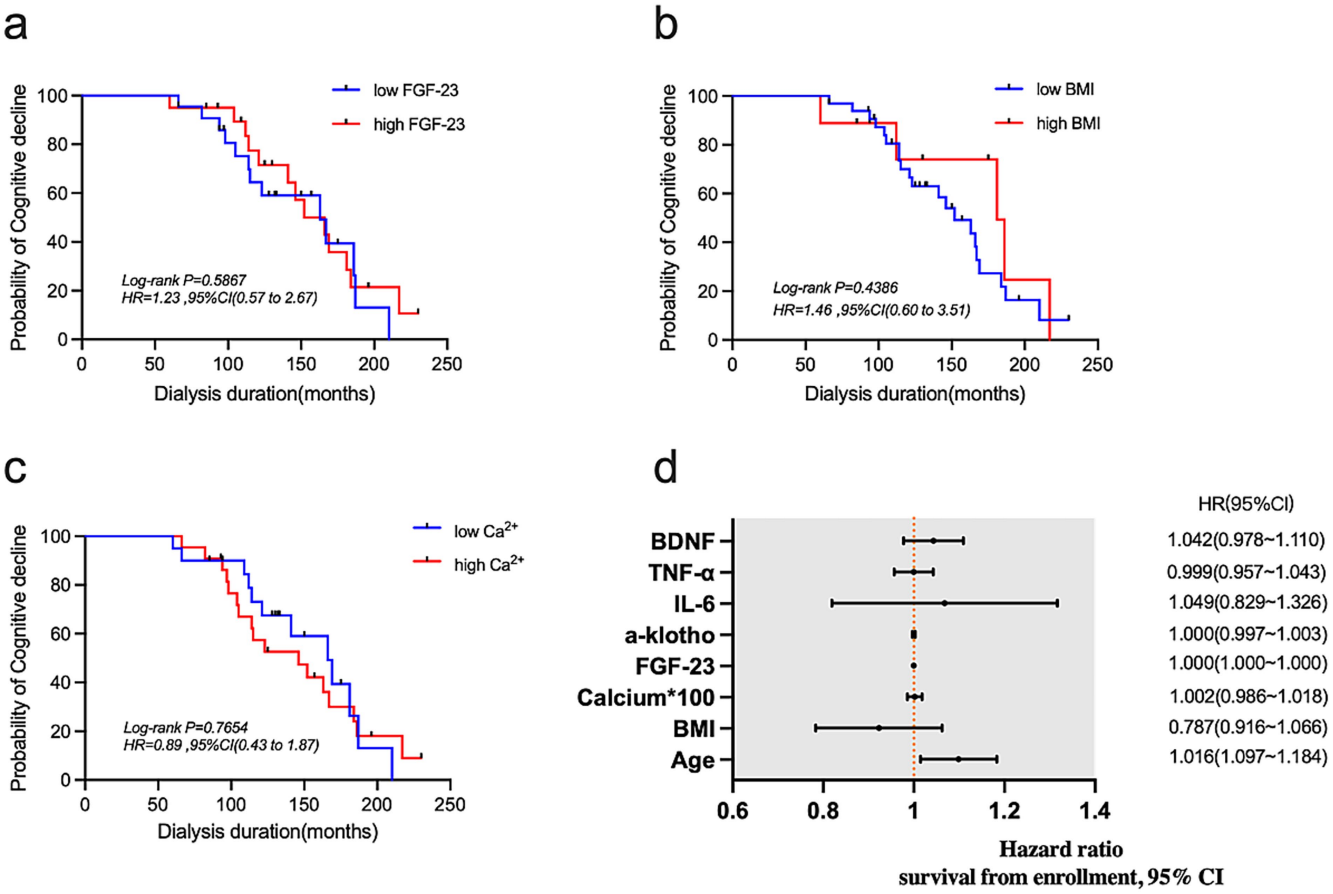
**(a–c)** Kaplan–Meier curves for FGF-23, BMI, and calcium. **(d)** Forest plot showing results of multivariate Cox proportional hazard analysis.

Next, we used survival analysis to predict the effects of different levels of FGF-23 (Figure 3a), BMI (Figure 3b) and calcium (Figure 3c) on the occurrence of cognitive impairment after 4 years. According to the results of multivariate Cox proportional hazard, we found that increasing age was a risk factor for cognitive impairment (Figure 3d).

## Discussion

In our initial study in 2018, we concluded that the Montreal Cognitive Assessment (MoCA) is broader and more sensitive than the Mini-Mental State Examination (MMSE), as well as more reasonable for people with severe cognitive impairment. Therefore, for this re-evaluation of cognitive function in maintenance hemodialysis (MHD) patients, we chose to use MoCA, considering its wider availability and affordability compared to other common cognitive impairment screening tests (6). The results of our study showed a significantly higher prevalence of cognitive impairment, at 69.0%, compared to 61.9% 4 years ago.

Based on the analysis of baseline information, we found that the age and dialysis age of patients 4 years later compared to 4 years ago were significantly different (*p* < 0.05). Advanced age is an independent risk factor for the onset of cognitive impairment (10–14), with cognitive change being a normal aging process. Some cognitive abilities, such as vocabulary, may improve with age, while reasoning, memory capacity, and processing speed decline over time (15). The occurrence of cognitive impairment is mainly observed in elderly patients due to common age-related diseases, such as atherosclerosis and hypertension, which can lead to cerebral blood deficiency and brain cell death, thereby affecting cognitive function. In addition, the hemodialysis treatment for patients with end-stage renal disease has been shown to effectively prolong survival. However, it is associated with potential adverse brain reactions such as transient ischemic attack (TIA) due to fluctuations in blood volume, blood pressure, and electrolyte levels during the process (16). We believe that the likelihood of iatrogenic adverse brain events increases with the age and duration of dialysis, resulting in a higher incidence of cognitive impairment. Therefore, elderly hemodialysis patients have a higher prevalence of cognitive impairment compared to the general elderly population.

Studies have shown that in non-diabetic dialysis patients, fat mass increases with age and muscle mass decreases (17). The increase of fat mass was indirectly related to the atrophy of frontal lobe, temporal lobe, and hippocampus (18). The hippocampus, which has key functions in memory, spatial level, and cognition (19), is recognized as a potential early biomarker for early memory decline and clinical dementia due to its reduced volume. In this study, we observed that the average BMI of 42 patients increased 4 years later compared to 4 years ago, with statistical differences (*p* < 0.05). Patients with high BMI also have a higher risk of cognitive impairment during dialysis, 2.28 times higher than those with low BMI. Thus, we propose that the increase in BMI among MHD patients is the result of increased fat mass, which can be further explored through human component analysis.

It is worth noting that the average blood calcium levels of 42 patients decreased 4 years later compared to 4 years ago, with statistical differences (*p* < 0.05). We also found in the survival curve that patients with low blood calcium levels were 1.31 times more at risk of cognitive impairment than those with high levels. This decrease in blood calcium levels among MHD patients could be attributed to various factors, including vitamin D deficiency, hypothyroidism, and the usage of calcium-containing phosphorus binders, vitamin D receptor agonists, and calcium-sensitive receptor agonists. The absorption of calcium is greatly affected by the prevalence of vitamin D deficiency in MHD patients. Vitamin D has various benefits for brain health, such as protecting neurons and having anti-inflammatory and anti-oxidative effects (20). Study has shown that vitamin D can maintain and enhance hippocampal synaptic function, preventing the decline of cognitive function, and vitamin D deficiency can potentially accelerate age-related cognitive decline (21). However, in this study, we did not measure the levels of vitamin D in MHD patients, and further research is needed to investigate the role of vitamin D in hemodialysis. Additionally, patients with hypothyroidism are more susceptible to intracranial vascular calcification. It is widely known that cognitive decline in aspects such as memory, emotion, execution, and spatial cognition is closely associated with intracranial and extracranial arterial stenosis caused by vascular calcification (22).

FGF-23, a serum biomarker, plays a crucial role in maintaining phosphate homeostasis and is synthesized and secreted by bone cells. In this study, we examined the serum biomarkers of patients with and without cognitive decline 4 years ago. The average FGF-23 levels were found to be higher in patients with cognitive decline, indicating a significant difference (*p* < 0.05). Our findings suggest a potential relationship between cognitive impairment in MHD patients and FGF-23 levels. The ROC curve analysis further revealed that FGF-23 has diagnostic value for cognitive impairment. But in the survival curve, we found that patients with high FGF-23 levels have a lower risk of cognitive decline later in life. It is not consistent with our previous study, and we may consider it because of the small sample size of the study and the large fluctuation range of FGF-23. The level of FGF-23 progressively increases in CKD patients as renal function deteriorates, aiming to maintain serum phosphate levels. In end-stage renal disease, FGF-23 levels may be up to 1,000 times higher than normal (23), which is consistent with previous research results (24). Li et al. (25) demonstrated that high phosphorus is a risk factor for dementia. Due to incomplete removal of excess phosphorus through hemodialysis treatment, MHD patients experience increased phosphorus deposition in the body, leading to a higher risk of cardiovascular and cerebrovascular diseases as well as varying degrees of neuropsychiatric or cognitive impairment (26). FGF-23 has also been found to play a significant role in neuronal morphology and synaptic density (27). Experimental studies have shown that FGF-23 can induce hippocampal atrophy and directly damage the cognitive function of CKD patients through its toxic effect on hippocampal neurons and synapses (28). The physiological role of FGF-23 is primarily mediated through the classic klotho-dependent pathway. Reduced expression of klotho in CKD patients increases the risk of cognitive decline as high klotho expression is considered neuroprotective (29, 30). Additionally, FGF-23 inhibits the activity of 1-α hydroxylase, leading to decreased vitamin D levels (31), which can further impact cognitive function.

Neuroinflammation is increasingly recognized as a contributing factor in neurodegenerative diseases, with numerous studies reporting a relationship between proinflammatory cytokines, such as tumor necrosis factor (TNF-α) and interleukin-6 (IL-6), and dementia and cognitive impairment (32–35). IL-6 plays a dual role by indirectly stimulating central nervous system inflammation through the blood–brain barrier and influencing cognition through its effect on cardiovascular disease (36, 37). Higher levels of IL-6 are thought to be associated with poorer cognitive function, while TNF-α. It indirectly affects cognitive function through macrophage mediated release of IL-6 (38). Brain-derived neurotrophic factor (BDNF) is widely distributed in the central nervous system, particularly in the hippocampus and cerebral cortex (39). BDNF impacts neuron survival, function, synaptic plasticity, long-term memory, and indirectly influences cognitive function by mediating neuroinflammation (40). While IL-6 and TNF-α levels are thought to negatively correlate with cognitive function, BDNF levels are positively correlated. Nonetheless, in our study, diagnostic accuracy of α-klotho, IL-6, TNF-α, and BDNF is low, and there is no statistical difference between the cognitive decline and cognitive undiminished groups. We attribute this to the small sample size of the study.

## Conclusion

Age, dialysis age, increased BMI, decreased blood calcium, and the abnormal level of FGF-23 may be independent risk factors for cognitive impairment in hemodialysis patients. BMI and decreased blood calcium can be used as predictive factors of hemodialysis-related cognitive impairment. Therefore, a longer follow-up study on the prognosis of MHD patients participating in this experiment is needed to further explore the effects of age, dialysis age, BMI, blood calcium, and the level of FGF-23 on cognitive impairment in MHD patients. Detection, diagnosis, and treatment should be prioritized in the clinic to prevent and delay the occurrence and development of cognitive impairment in MHD patients, thereby improving their quality of life.

## Data Availability

The original contributions presented in the study are included in the article/supplementary material, further inquiries can be directed to the corresponding authors.
